# Hepatitis C Virus (HCV)—Mediated Activation of Hexokinase Domain-Containing Protein 1 (HKDC1) Promotes Hexokinase Activity and Metabolic Reprogramming

**DOI:** 10.3390/v18040423

**Published:** 2026-03-31

**Authors:** Hope K. Fiadjoe, Amani Doyle, In-Woo Park, Pankaj Chaudhary

**Affiliations:** Department of Microbiology, Immunology, and Genetics, University of North Texas Health Science Center, Fort Worth, TX 76107, USA; hopefiadjoe@my.unthsc.edu (H.K.F.); amanidoyle@my.unthsc.edu (A.D.); inwoo.park@unthsc.edu (I.-W.P.)

**Keywords:** HCV, HKDC1, hexokinase, glycolysis, metabolic reprogramming

## Abstract

Hepatitis C virus (HCV) infection is a significant contributor to the development of hepatocellular carcinoma (HCC). One mechanism by which HCV promotes HCC is the remodeling of host cell metabolism; however, the molecular mediators of this process are not yet fully understood. In this study, we identified Hexokinase Domain-Containing Protein 1 (HKDC1) as a crucial effector that links HCV infection to glycolytic reprogramming in hepatoma cells. HCV-positive APC140 cells showed selective upregulation of HKDC1, accompanied by enhanced cytoplasmic localization of the protein. Moreover, these cells exhibited increased total hexokinase activity and elevated pyruvate and lactate production, while the classical hexokinases HK1, HK2, HK3, and HK4 remained unchanged. Depleting HKDC1 led to a reduction in hexokinase activity, glycolytic flux, and HCV subgenomic replicon-associated reporter activity, with no compensatory changes noted in other members of the hexokinase family. These findings indicate that HCV-induced HKDC1 creates a metabolic environment conducive to viral replication and may contribute to HCC development. Therefore, HKDC1 acts as a virus-responsive metabolic mediator that links chronic HCV infection to oncogenic metabolic reprogramming, positioning it as a potential therapeutic target in HCV-associated HCC.

## 1. Introduction

Hepatocellular carcinoma (HCC) is the most common primary liver malignancy and a leading cause of cancer-related mortality worldwide [1]. Its high mortality is largely attributed to late clinical presentation, underlying liver dysfunction, and limited therapeutic options in advanced disease [1,2]. Chronic hepatitis C virus (HCV) infection remains a major etiological factor for HCC, driving progressive liver injury, cirrhosis, and malignant transformation through persistent inflammation, oxidative stress, and sustained metabolic alterations [3,4,5]. Approximately 2.4 million people in the United States and 71 million people in the world have chronic hepatitis C. HCC caused by HCV infection has a higher risk of recurrence compared with non-viral or HBV-induced HCC [6]. HCV infection is associated with increased expression of pro-metastatic factors in patients with HCC. HCV infection has significantly higher intrahepatic and extrahepatic metastatic dissemination of HCC cells compared with non-viral HCC [7]. Although the use of direct-acting antivirals has markedly improved viral clearance, HCV-associated metabolic reprogramming and oncogenic risk may persist even after viral eradication [8].

Metabolic reprogramming is a hallmark of HCC, reflecting the high bioenergetic and biosynthetic demands of tumor cells [9]. Enhanced glucose uptake and altered glycolytic flux support rapid proliferation, redox balance, and survival under hypoxic and nutrient-limited conditions [10]. HCV replication is dependent on host cellular metabolism, particularly pathways involved in glucose utilization. Metabolic alterations in HCV-infected hepatocytes are required to support viral replication. These virus-induced metabolic changes overlap with metabolic features characteristic of liver tumorigenesis, making glucose metabolism a critical factor in viral persistence and HCC development. Experimental studies have shown that HCV enhances glycolysis primarily by modulating enzyme activity rather than protein expression. For example, NS5A, a non-structural protein of HCV, directly binds with hexokinase 2 (HK2) and increases glycolytic flux by altering catalytic parameters in HCV-infected Huh7.5 and Huh9.13 subgenomic replicon cells [11]. NS5A also activates hepatic glucokinase, which increases glucose utilization and redirects carbon flux toward lipogenesis [12]. However, HK2 expression is not consistently elevated in HCV-related HCC, unlike in hepatitis B virus-related tumors [13]. This suggests that canonical HK2-driven metabolic changes do not fully explain the metabolic regulation in HCV-associated disease and that other hexokinase family members may play a role.

Hexokinase domain-containing protein 1 (HKDC1) is a recently identified hexokinase-like protein and a proposed fifth member of the hexokinase family. The HKDC1 gene is located on chromosome 10 and is adjacent to HK1; it encodes a ~100 kDa protein with ~70% sequence similarity to HK1 [14,15,16]. HKDC1 was first discovered through a genome-wide association study linking it to gestational glucose homeostasis [17]. It is expressed in the liver, kidney, brain, and retina, and is upregulated under metabolic and stress conditions such as pregnancy, aging, and hepatic stress [18,19]. Similar to HK1 and HK2, HKDC1 associates with the outer mitochondrial membrane via interaction with the voltage-dependent anion channel (VDAC) [14]. It contributes to whole-body glucose homeostasis [19] and has been reported to maintain mitochondrial and lysosomal homeostasis and prevent cellular senescence [20].

Aberrant HKDC1 expression has been reported in multiple malignancies, including hepatocellular carcinoma, and ocular disorders. Across cancer types, HKDC1 has been shown to promote tumor growth and aggressive phenotypes. Mechanistically, it enhances glucose consumption, activates AMPK/mTOR signaling, interacts with VDAC1 to regulate mitochondrial function, supports cancer cell survival, and contributes to chemoresistance. High HKDC1 expression is associated with immune evasion and poor prognosis, including shorter overall survival [15,16,21,22,23,24,25,26,27,28,29,30,31]. In the liver, HKDC1 is nearly undetectable in normal tissue but is specifically upregulated in malignant cells of HCC and cholangiocarcinoma [32]. Under hypoxic conditions, HKDC1 promotes cancer stemness [32]. HKDC1 is implicated in non-alcoholic fatty liver disease, non-alcoholic steatohepatitis, and liver fibrosis, with elevated expression particularly in NASH [19]. In alcoholic hepatitis, HKDC1 is the most upregulated kinase and contributes to dysregulated glucose metabolism [33]. In HCC, high HKDC1 levels correlate with poor prognosis and aggressive phenotypes [32,34], and HKDC1 promotes proliferation and migration through the Wnt/β-catenin pathway [32,34]. Silencing HKDC1 in HCC cell lines, such as HepG2, suppresses proliferation and migration and downregulates β-catenin and c-Myc [32]. HKDC1 also shapes the tumor immune microenvironment by activating the STAT1/PD-L1 axis, which promotes immune evasion and may contribute to resistance to therapy [35]. HKDC1 appears to be the most tumor-cell-specific hexokinase in primary liver cancer, especially in cases with distant metastases [32].

The metabolic roles of HKDC1 are particularly relevant in the liver, where chronic metabolic stress and viral infection converge to drive disease progression. Given that HCV replication is highly dependent on host glycolytic pathways, these findings raise the possibility that HKDC1 can function as a metabolic link between HCV infection and oncogenic programming in hepatocytes. A previous transcriptomic analysis identified HKDC1 as a distinct molecular signature that is differentially expressed across stages of HCV-related liver disease [36]. In HCV HCC subgenomic replicon models, HKDC1 levels increased at both the mRNA and protein levels, indicating responsiveness to viral replication [37,38]. Despite these observations, the functional role of HKDC1 in HCV-driven metabolic remodeling remains unclear.

This study investigates how HKDC1 contributes to glucose metabolism in HCV-associated HCC. We integrate functional assays, including hexokinase activity, lactate, and pyruvate production, to assess the impact of HKDC1 knockdown in an HCV replicon system. Our goal is to define how HKDC1 influences metabolic reprogramming in the context of HCV-driven liver cancer.

## 2. Materials and Methods

### 2.1. Cell Lines and Culture Conditions

The human hepatoma cell line Huh7.5.1 and a derivative subgenomic hepatitis C virus (HCV) replicon cell line, APC140, were used in this study. APC140 cells contain a JFH1 strain-derived subgenomic replicon expressing viral regulatory genes and a Renilla luciferase reporter. Both cell lines were obtained from Apath, L.L.C. (New York, NY, USA). Cells were cultured in low-glucose Dulbecco’s Modified Eagle’s Medium (DMEM containing 1 g/L glucose; Corning, NY, USA) supplemented with 10% fetal bovine serum (GIBCO, Waltham, MA, USA). To maintain replicon stability, APC140 cells were cultured in the presence of 0.5 g/L G418 (Thermo Scientific, Waltham, MA, USA), whereas Huh7.5.1 cells were maintained without antibiotic selection. All cultures were incubated at 37 °C in a humidified atmosphere containing 5% CO_2_.

### 2.2. Knockdown of HKDC1 in APC140 Cells

Plasmids containing scrambled control shRNA (Catalog number: CSHCTR001-LVRU6GP) and human HKDC1 shRNA (Catalog number: HSH019581-LVRU6GP) were obtained from GeneCopoeia, Inc. (Rockville, MD, USA). Lentivirus was packaged in HEK293T cells using the packaging plasmid VSVG and the helper plasmid Δ8.9. Subsequently, the virus was transduced into APC140 cells using 5 µg/µL polybrene. APC140 cells were then selected with puromycin (2 µg/mL, Gibco, Waltham, MA, USA) for stable HKDC1 knockdown cells. Immunoblot analysis was performed to confirm HKDC1 knockdown in APC140 cells.

### 2.3. Reporter Gene Assay

Luciferase reporter gene assay was performed, using the Renilla Luciferase assay kit (Promega Corporation, Madison, WI, USA), according to the manufacturer’s protocol. Briefly, HCV subgenomic replicon cells, APC140, were washed twice with PBS and lysed in Renilla luciferase lysis buffer. The luciferase activity in the lysates was measured in triplicate using a GloMax Multiplus Plate Reader (Promega Corporation, Madison, WI, USA). For comparison, Huh7.5.1 cells were used as a negative control. This assay was also performed in APC140 cells expressing either a non-targeting shRNA or an HKDC1-targeting shRNA construct.

### 2.4. RNA Extraction and Quantitative Real-Time Polymerase Chain Reaction (qRT-PCR) Analysis

Total cellular RNA was extracted using TRIzol reagent (Invitrogen, Waltham, MA, USA). RNA was quantified using a NanoDrop 2000 instrument (Thermo Scientific, Waltham, MA, USA). Reverse transcription was carried out on 1 μg of RNA using TaqMan reverse transcription reagents (Applied Biosystems, Foster City, CA, USA) according to the manufacturer’s instructions. Then, 40 cycles of PCR were performed with 1 μL of RT product using THUNDERBIRD Next SYBR qPCR Mix (Toyobo Co., Ltd., Osaka, Japan) in the presence of 50 pmol of sense- and antisense-specific primers for HK1 (forward primer: 5′-CTGCTGGTGAAAATCCGTAGTGG-3′; reverse primer: 5′-GTCCAAGAAGTCAGAGATGCAGG-3′), HK2 (forward primer: 5′-GAGTTTGACCTGGATGTGGTTGC-3′; reverse primer: 5′-CCTCCATGTAGCAGGCATTGCT-3′), HK3 (forward primer: 5′-CATCGTGGACTTCCAGCAGAAG-3′; reverse primer: 5′-CTTGGTCCAGTTCAGGAGGATG-3′), HK4 (forward primer: 5′-CATCTCCGACTTCCTGGACAAG-3′; reverse primer: 5′-TGGTCCAGTTGAGAAGGATGCC-3′), HKDC1 (forward primer: 5′-ATCGCCGACTTCCTGGACTACA-3′; reverse primer: 5′-GCCTTGAAACCTTTGGTCCACC-3′), or β-actin (forward primer: 5′-CACCATTGGCAATGAGCGGTTC-3′; reverse primer: 5′-AGGTCTTTGCGGATGTCCACGT-3′) in accordance with the manufacturer’s instructions. PCR was carried out as follows: 95 °C for 10 min followed by 40 cycles of 95 °C for 15 s and 60 °C for 60 s. β-actin was used as an endogenous control to normalize the data. All quantitative real-time PCR experiments were performed in triplicate.

### 2.5. Immunoblot Analysis

Monolayers of cells were washed in cold phosphate-buffered saline (PBS) and lysed in RIPA lysis buffer containing protease and phosphatase inhibitor cocktails (Millipore Sigma, Burlington, MA, USA) on ice for 5 min. Lysates were collected by scraping, transferred to 1.5 mL tubes, sonicated, and insoluble debris was pelleted by centrifugation at 15,000× *g* for 10 min at 4 °C. The supernatant was transferred to a new tube, and protein concentration was determined using the Pierce BCA Protein Assay Kit (Thermo Scientific, Waltham, MA, USA). Equal amounts of protein were separated by SDS-PAGE and analyzed by immunoblotting on a nitrocellulose membrane. Membranes were blocked for 1 h in 5% non-fat milk dissolved in Tris-buffered saline, pH 7.5, with 0.1% Tween-20 (TBST). Membranes were incubated overnight at 4 °C in primary antibodies for anti-HK1 (ABclonal #A0533), anti-HK2 (ABclonal #A22319), anti-HK3 (Invitrogen #PA5-15441), anti-HK4/GCK (ABclonal #A6293), anti-HKDC1 (Abcam #AB209846), anti-NS5A (Abcam #ab65410), anti-HCV NS5B (ViroGen #266-A), Na^+^/K^+^ ATPase alpha-1 (BioLegend #604751), anti-Lamin A/C (ABclonal Technology #A19524), β-actin (Santa Cruz Biotechnology #sc47778), or anti-GAPDH (Santa Cruz Biotechnology #sc-32,233). After primary antibody incubation, membranes were washed for 10 min with TBST three times. The membranes were then incubated with anti-mouse IgG (Southern Biotechnology) or anti-rabbit IgG (Southern Biotechnology, Birmingham, AL, USA) secondary antibody in 5% non-fat milk in TBST for 2 h at room temperature. The membranes were rinsed for 10 min with TBST three times, then imaged with Immobilon Western Chemiluminescent HRP Substrate (Millipore Sigma) on an Azure 300 (Azure Biosystems, Dublin, CA, USA) imager. The intensity of protein bands was quantified by densitometry using ImageJ software (version 1.54p; NIH, Bethesda, MD, USA).

### 2.6. Subcellular Fractionation

Subcellular fractionation was performed to assess HKDC1 localization in Huh7.5.1 and APC140 cell lines. Cells were harvested on ice and fractionated into cytoplasmic, membrane/organelle, and nuclear compartments using a subcellular protein fractionation kit (Thermo Fisher Scientific #78840) according to the manufacturer’s protocol. Protein concentrations were determined using the BCA assay and resolved by SDS-PAGE. Proteins were transferred to nitrocellulose membranes and probed for HKDC1 using immunoblot analysis. Fraction purity was confirmed using compartment-specific marker proteins.

### 2.7. Immunofluorescence Studies

Huh7.5.1 and APC140 cells were cultured to 50% confluence on glass coverslips in 12-well plates. Cells were fixed using 4% paraformaldehyde and subsequently permeabilized with 0.1% Triton X-100 for 20 min. Following this, the coverslips were washed with PBS and incubated in 5% goat serum in PBS for 2 h. The coverslips were incubated with anti-HKDC1 antibody at a dilution of 1:100 in PBS overnight at 4 °C. After further washing with PBS, the coverslips were incubated with Alexa Fluor 488 goat anti-rabbit IgG (Life Technologies, Carlsbad, CA, USA) at a dilution of 1:400 in PBS for 2 h at room temperature in the dark. The coverslips were then washed with PBS and mounted onto glass slides using VectaShield vibrance antifade mounting medium with DAPI (Vector Laboratories #H-1800). The slides were examined using an LSM 880 confocal system (Carl Zeiss, Jena, Germany) equipped with an inverted microscope (Axio Observer 7, Carl Zeiss, Jena, Germany).

### 2.8. Lactate and Pyruvate Assay

Extracellular lactate and pyruvate levels were measured using the Lactate-Glo™ Assay (#J5021, Promega Corporation, Madison, WI, USA) and Pyruvate-Glo™ Assay (#J4051, Promega Corporation, Madison, WI, USA), respectively, according to the manufacturer’s protocol. Luminescence (RLU) was recorded using a Synergy HT microplate reader (BioTek, Winooski, VT, USA).

### 2.9. Hexokinase Activity Assay

Hexokinase activity was measured using the hexokinase activity assay kit (#AB136957, Abcam Limited, Cambridge, MA, USA) according to the manufacturer’s instructions. Cells were seeded in triplicate in 6-well plates. When cells reached 80% confluency, they were washed with cold PBS on ice and harvested in an ice-cold assay buffer by scraping. Cells were homogenized with a Dounce homogenizer (Fisher Scientific, Waltham, MA, USA) at 4 °C, lysates were centrifuged for 10 min at 12,000× *g* at 4 °C, and supernatants were collected. Total cellular protein was measured in supernatants by BCA and was used for normalization. Samples (40 µg protein) were diluted in assay buffer. The reaction was initiated by adding a reaction mix containing HK substrate, ATP, G-6-P dehydrogenase, and NADP+, followed by incubation at room temperature for 30 min. The resulting colorimetric signal was measured at 450 nm using a microplate reader.

### 2.10. Statistical Analysis

Data are presented as mean ± SEM of the indicated independent experiments. Statistical analysis was performed using the unpaired two-tailed Student’s *t*-test for comparison between two groups. *p*-values less than 0.05 were considered statistically significant.

## 3. Results

### 3.1. HCV Infection Enhances Hexokinase Activity, Lactate, and Pyruvate Production

Given that HCV replication is highly dependent on host glucose metabolism [39,40,41], we first assessed whether the APC140 subgenomic replicon model exhibits a glycolytic phenotype. Total hexokinase activity and the production of key glycolytic metabolites, pyruvate and lactate, were measured to establish the metabolic state of HCV-positive cells relative to the parental controls.

**Figure 1:**
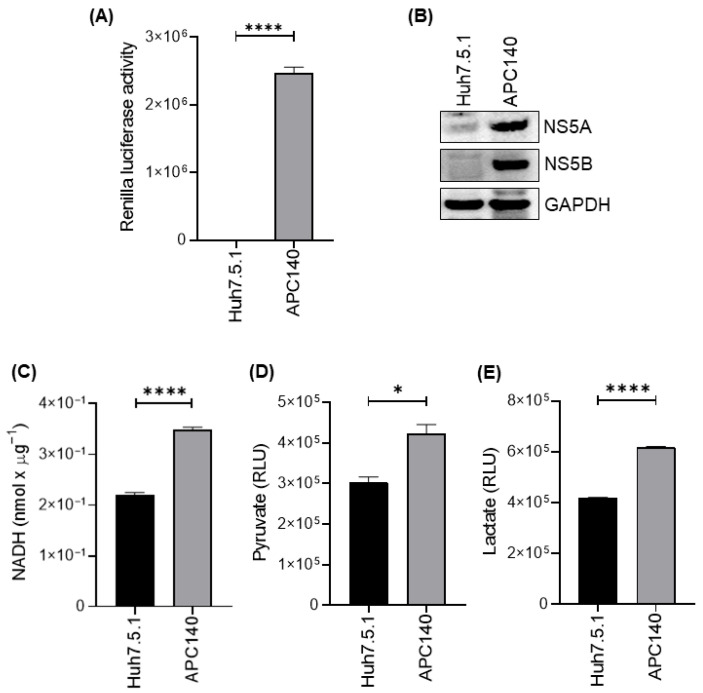
HCV subgenomic replicon increases hexokinase activity and glycolytic metabolites in hepatoma cells. (**A**) Bar graph showing Renilla luciferase reporter activity in parental Huh7.5.1 (HCV-negative) and APC140 (HCV replicon-positive) cells. Data are expressed as mean ± SEM (**** *p*-value < 0.0001; unpaired two-tailed Student’s *t*-test). (**B**) Immunoblot analysis detecting the expression of HCV non-structural viral proteins NS5A and NS5B in APC140 cells, but not in Huh7.5.1 cells. GAPDH was used as a loading control. (**C**) Bar graph showing hexokinase activity in the homogenates of Huh7.5.1 and APC140 cells measured using an NADH-coupled enzymatic assay. Data are expressed as the mean ± SEM (**** *p*-value < 0.0001; unpaired two-tailed Student’s *t*-test). (**D**) Bar graph showing extracellular pyruvate levels (RLU), demonstrating a significant increase in APC140 cells relative to Huh7.5.1 cells (* *p* < 0.05; Student’s *t*-test). (**E**) Bar graph showing extracellular lactate levels (RLU), significantly elevated in APC140 cells compared with Huh7.5.1 (**** *p* < 0.0001; unpaired two-tailed Student’s *t*-test). Uncropped immunoblots of (**B**) are available in Supplementary File S1.

Accordingly, the first goal of this study was to validate whether the APC140 subgenomic model exhibits active HCV replicon activity and a corresponding glycolytic phenotype before interrogating specific mediators. In this study, Renilla luciferase reporter activity was significantly higher in APC140 cells than in Huh7.5.1 cells (Figure 1A), consistent with active HCV replication in this model system. Immunoblot analysis further confirmed HCV replication in APC140 cells by robust expression of the non-structural viral proteins NS5A and NS5B. These viral proteins were not detected in parental Huh7.5.1 cells (Figure 1B), validating the HCV-positive status of APC140 cells.

We next assessed total cellular hexokinase activity using an NADH-coupled enzymatic assay. HCV-positive APC140 cells exhibited a significant increase in total hexokinase activity compared to HCV-negative Huh7.5.1 cells (Figure 1C). Additionally, HCV-positive APC140 cells demonstrated significantly elevated levels of pyruvate and lactate production, as measured by a luminescence-based assay (Figure 1D and Figure 1E, respectively). These findings suggest that HCV contributes to the induction of glycolysis in APC140 cells that are actively replicating the virus.

### 3.2. HCV Infection Selectively Promotes HKDC1 Expression at mRNA and Protein Levels

Given the increase in total hexokinase activity observed in APC140 cells, we investigated whether HCV alters the expression of hexokinases. Hexokinases catalyze the first step of glucose metabolism by phosphorylating glucose, thereby trapping it intracellularly and controlling its entry into glycolysis and related anabolic pathways [15]. While canonical isoforms (HK1–HK3 and glucokinase/HK4) have been well studied, HKDC1 is increasingly recognized as a fifth hexokinase-like protein with measurable hexokinase activity and broad tissue expression, and its dysregulation has been implicated in metabolic stress and cancer biology [14,42]. Because tumor-like metabolic adaptation can involve the selective use of particular glycolytic entry enzymes, identifying the specific isoform driving increased total hexokinase activity is essential.

**Figure 2:**
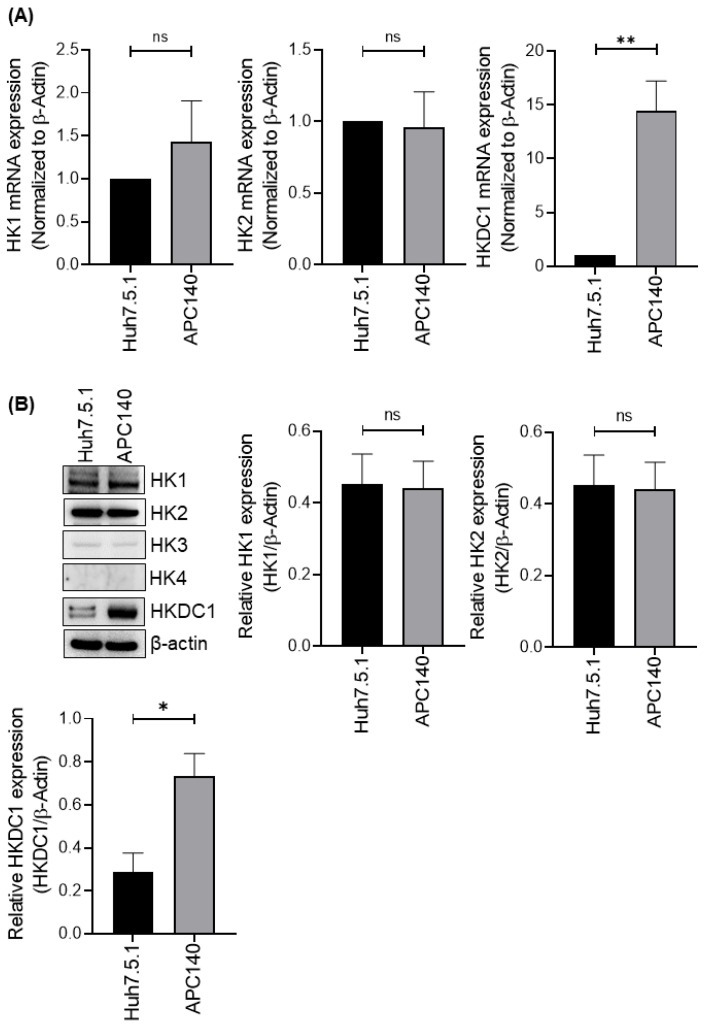
HCV infection selectively induces HKDC1 expression. (**A**) Bar graph showing qRT-PCR quantification of HK1, HK2, and HKDC1 mRNA in Huh7.5.1 and APC140 cells. HKDC1 mRNA was significantly increased in APC140 cells (ns, not significant; ^ns^ *p* ˃ 0.05; ** *p* < 0.01; unpaired two-tailed Student’s *t*-test), whereas HK1 and HK2 remained unchanged; HK3 and HK4 transcripts were undetectable. Expression was normalized to β-actin. (**B**) Immunoblot showing HKDC1 protein levels in Huh7.5.1 and APC140 cells, with densitometric analysis confirming a significant increase in HKDC1 expression in APC140 (^ns^ *p* ˃ 0.05; * *p* < 0.05; unpaired two-tailed Student’s *t*-test). β-actin serves as a loading control. Uncropped immunoblots of (**B**) are available in Supplementary File S1.

Therefore, we quantified the mRNA and protein expression levels of the major hexokinase family members to determine whether the increased activity reflected the induction of classical isoforms or a more selective shift. Quantitative PCR analysis revealed no significant changes in HK1 or HK2 expression between HCV-positive APC140 and HCV-negative Huh7.5.1 cell lines (Figure 2A), whereas HK3 and HK4 were undetectable. In contrast, HKDC1 mRNA expression was significantly increased in APC140 cells compared with that in Huh7.5.1 cells (Figure 2A). Consistent with the transcriptional data, immunoblot analysis showed a significant increase in HKDC1 protein levels in APC140 cells compared with those in parental Huh7.5.1 cells (Figure 2B). However, there was no change in the expression of HK1 and HK2, whereas HK3 and HK4 were undetectable at the protein level (Figure 2B). Collectively, these results indicate that HCV infection increases HKDC1 expression at both the mRNA and protein levels in hepatoma cells.

### 3.3. HCV Infection Enhances Cytoplasmic Accumulation of HKDC1 in HCV-Positive HCC Cells

Subcellular localization is a functional determinant of glucose-metabolic enzymes because proximity to key substrates and organellar interfaces shapes metabolic flux and signaling outputs. HKDC1 is primarily localized to the outer mitochondrial membrane within the cytoplasm, where it interacts with voltage-dependent anion channels to couple glucose metabolism with mitochondrial function and support metabolic reprogramming in liver cancer cells [14,43]. Further report by the Human Protein Atlas shows that HKDC1 is predominantly cytoplasmic with a granular distribution across multiple human tissues. In addition to its canonical metabolic functions, HKDC1 has been reported to be nuclear-localized, where it phosphorylates chromatin regulators, influencing gene expression and cell proliferation in hepatocellular carcinoma [44]. Localization changes can be driven by stress, infection, metabolic cues, or oncogenic signaling [45]. Viruses, including the hepatitis C virus, rewire host metabolism not only by altering gene expression but also by reorganizing the subcellular positioning of key metabolic enzymes [46]. Therefore, in addition to measuring abundance, determining whether HCV shifts HKDC1 to cellular compartments compatible with glycolytic control provides key functional support for HKDC1 as a mediator of HCV-associated metabolic remodeling.

**Figure 3:**
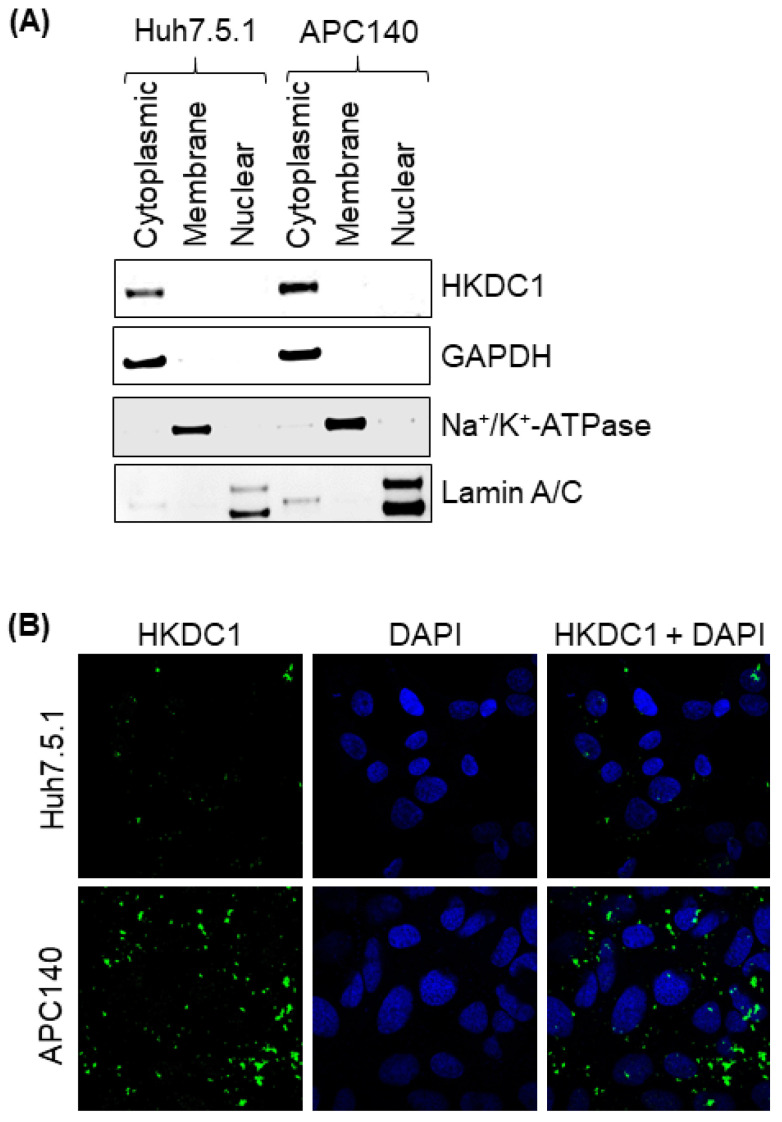
HCV subgenomic replicon promotes cytoplasmic accumulation of HKDC1. (**A**) Immunoblots from subcellular fractionation showing increased HKDC1 levels in the cytoplasmic fraction of APC140 cells relative to Huh7.5.1 cells. GAPDH, Na^+^/K^+^ ATPase, and Lamin A/C served as markers for the cytoplasmic, membrane, and nuclear fractions, respectively. (**B**) Immunofluorescence staining of HKDC1 in Huh7.5.1 and APC140 cells, showing increased expression of HKDC1 in the cytoplasmic fraction of APC140 cells. HKDC1 was detected using an anti-HKDC1 antibody, followed by an Alexa Fluor 488 IgG secondary antibody (green). Nuclei were stained with DAPI (blue), and images were acquired using a Zeiss LSM 880 confocal microscope. Uncropped immunoblot of (**A**) is available in Supplementary File S1.

To investigate whether HCV infection alters the subcellular distribution of HKDC1, we performed biochemical fractionation of parental Huh7.5.1 and HCV-positive APC140 cells, followed by immunoblot analysis of cytoplasmic, membrane, and nuclear fractions. In the cytoplasmic fraction, HKDC1 protein was detected at low levels in Huh7.5.1 cells but was substantially enriched in APC140 cells (Figure 3A). Fraction purity and integrity were validated using established compartment-specific markers—GAPDH for cytoplasmic fractions, Na^+^/K^+^-ATPase for membrane fractions, and Lamin A/C for nuclear fractions. To visually corroborate the subcellular fractionation data, HKDC1 localization was further examined using confocal immunofluorescence microscopy. Our results clearly demonstrated that HKDC1 was primarily localized to the cytoplasm, with minimal nuclear overlap. Notably, HCV-positive APC140 cells showed elevated cytoplasmic HKDC1 intensity relative to Huh7.5.1 controls (Figure 3B).

### 3.4. HKDC1 Depletion Impairs Glycolytic Activity in HCV-Infected HCC Cells

Although changes in expression suggest a candidate mechanism, causal inference requires functional perturbation. In the cancer and metabolic stress literature, HKDC1 has been linked to metabolic adaptation, and experimental depletion of HKDC1 can disrupt cellular energy homeostasis and mitochondrial function [43]. Thus, loss-of-function testing in the HCV replicon context is a critical step to determine whether HKDC1 mechanistically drives the glycolytic changes observed in APC140 cells.

**Figure 4:**
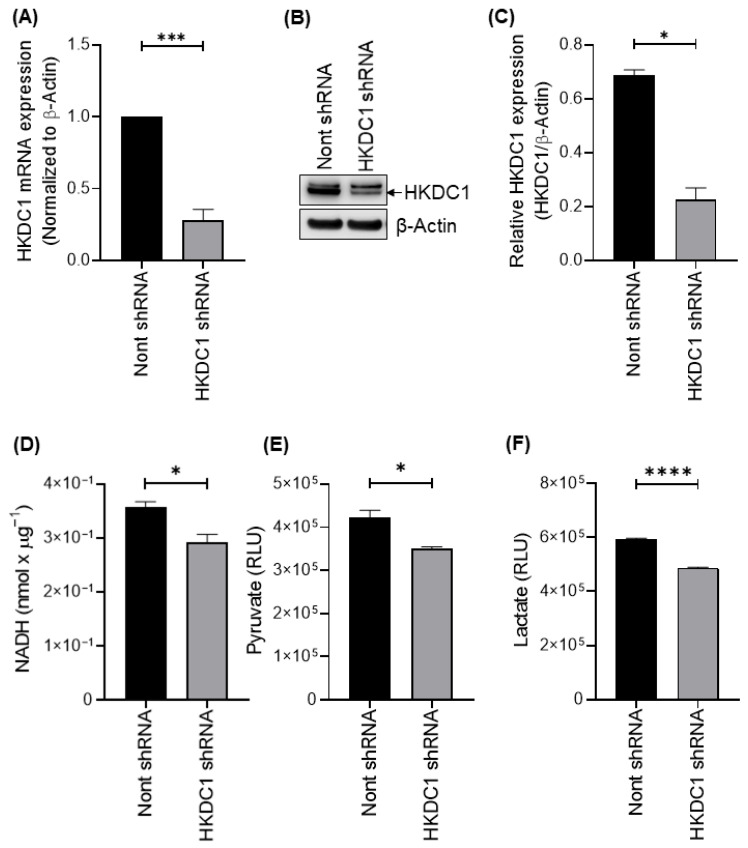
HKDC1 knockdown reduces hexokinase activity and glycolytic flux in HCV-infected cells. (**A**) Bar graph showing qRT-PCR confirmation of HKDC1 mRNA knockdown in APC140 cells expressing HKDC1-targeting shRNA compared with non-targeting shRNA control (*** *p* < 0.001; unpaired two-tailed Student’s *t*-test). (**B**) Immunoblot validating HKDC1 protein depletion in APC140 cells following shRNA treatment. β-actin is used as a loading control. (**C**) Densitometric bar graph quantifying HKDC1 protein levels from panel B, normalized to β-actin, showing a significant reduction (* *p* < 0.05; unpaired two-tailed Student’s *t*-test). (**D**) Bar graph showing reduced hexokinase activity in HKDC1-depleted APC140 cells compared to non-targeting shRNA control, measured via an NADH-coupled assay (* *p* < 0.05; unpaired two-tailed Student’s *t*-test). (**E**) Bar graph showing the significantly decreased extracellular pyruvate levels (RLU) in HKDC1-depleted APC140 cells compared to APC140 cells expressing non-targeting shRNA (* *p* < 0.05; unpaired two-tailed Student’s *t*-test). (**F**) Bar graph showing markedly decreased extracellular lactate levels (RLU) following HKDC1 knockdown (**** *p* < 0.0001; unpaired two-tailed Student’s *t*-test). Uncropped immunoblot of (**B**) is available in Supplementary File S1.

We used shRNA to perform targeted knockdown of HKDC1 mRNA and protein. Quantitative PCR analysis confirmed efficient depletion of HKDC1 mRNA in cells expressing HKDC1-targeting shRNA compared with non-targeting control shRNA (Figure 4A). Consistent with transcript-level suppression, immunoblot analysis demonstrated reduced HKDC1 protein abundance following HKDC1 knockdown (Figure 4B). Densitometric quantification, normalized to β-actin, confirmed a statistically significant reduction in HKDC1 protein levels (Figure 4C).

Following HKDC1 knockdown, we evaluated the effect of HKDC1 depletion on cellular metabolism. Total cellular hexokinase activity, measured using an NADH-coupled assay, was significantly reduced in HKDC1 knockdown cells relative to non-targeting shRNA controls (Figure 4D). In parallel, both pyruvate and lactate production were markedly decreased following HKDC1 knockdown, as assessed by the luminescence-based assays (Figure 4E and Figure 4F, respectively).

### 3.5. HKDC1 Depletion Does Not Affect the Expression of Other Hexokinases

Because a family of related enzymes controls glycolytic entry, compensatory regulation can obscure causal inferences when one enzyme is perturbed [47,48]. HKDC1 shares structural similarities with HK1 and is part of a broader hexokinase network, making it important to determine whether metabolic effects following HKDC1 depletion reflect a direct loss of HKDC1 function versus secondary adaptation through other isoforms [14]. Demonstrating a lack of compensatory induction strengthens the argument that HKDC1 is the key virus-responsive metabolic mediator in this setting.

**Figure 5:**
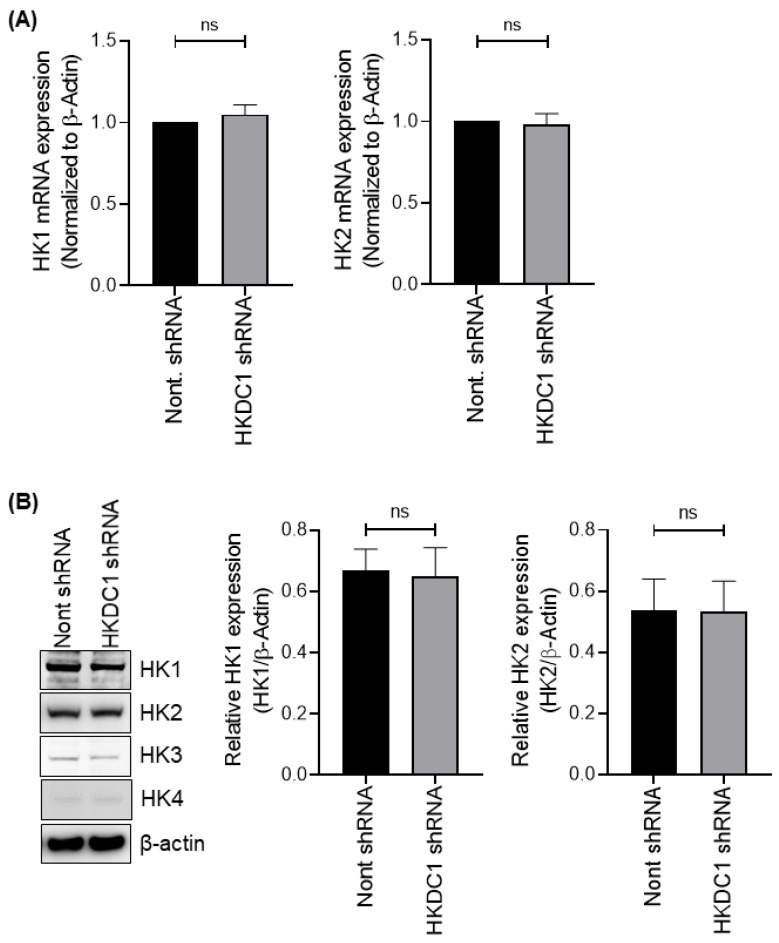
HKDC1 depletion does not alter the expression of other hexokinase isoforms. (**A**) Bar graphs showing qRT-PCR analysis of HK1 and HK2 mRNA levels in APC140 cells expressing non-targeting or HKDC1-targeting shRNA. Transcript levels remained unchanged following HKDC1 knockdown (ns, not significant; ^ns^ *p* ˃ 0.05, unpaired two-tailed Student’s *t*-test). HK3 and HK4 transcripts were undetectable. Expression was normalized to β-actin. (**B**) Immunoblot showing HK1, HK2, HK3, and HK4 protein expression in APC140 cells with or without HKDC1 depletion. β-actin was used as a loading control. (^ns^ *p* ˃ 0.05, unpaired two-tailed Student’s *t*-test). Uncropped immunoblots of (**B**) are available in Supplementary File S1.

Upon HKDC1 depletion, quantitative PCR analysis revealed no significant changes in the mRNA expression levels of HK1 or HK2 when comparing non-targeting shRNA to HKDC1 shRNA knockdown in HCV-positive APC140 cells (Figure 5A). Moreover, HK3 and HK4 were undetectable. In alignment with the transcriptional data, immunoblot analysis did not reveal any significant differences in the protein levels of HK1 and HK2 between the non-targeting shRNA and HKDC1 shRNA knockdown conditions in HCV-positive APC140 cells (Figure 5B), with HK3 and HK4 also remaining undetected. These findings suggest that the elevated hexokinase activity, as well as the production of pyruvate and lactate observed in HCV-positive APC140 cells, is attributable to the high expression of HKDC1 in these cells.

### 3.6. HKDC1 Knockdown Inhibits the Replication of HCV

**Figure 6:**
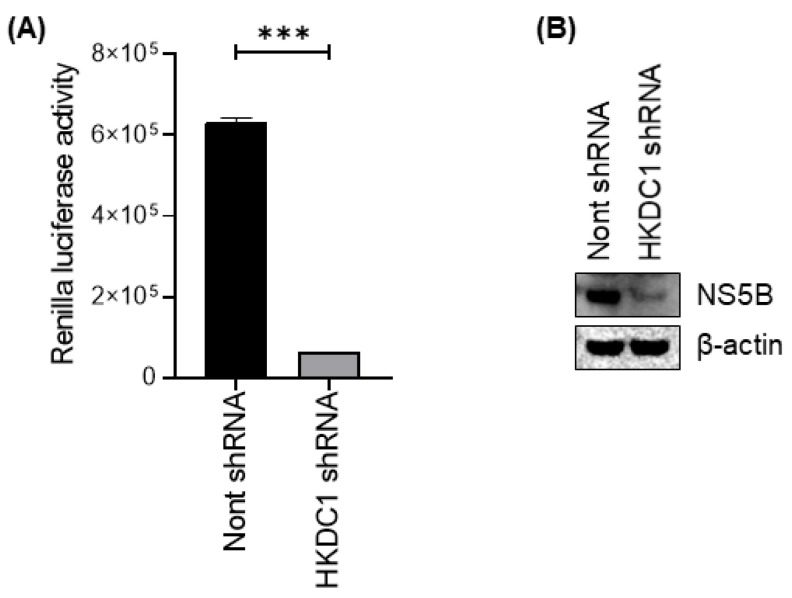
HKDC1 depletion reduces HCV replication and the expression of HCV non-structural proteins. (**A**) Bar graph showing Renilla luciferase activity, a surrogate for HCV RNA replication, in cells expressing non-targeting or HKDC1-targeting shRNA. HKDC1 knockdown caused a significant decrease in the reporter signal (*** *p* < 0.001; unpaired two-tailed Student’s *t*-test). (**B**) Immunoblot showing NS5B protein expression in APC140 cells expressing non-targeting (Nont) shRNA or HKDC1 shRNA. β-actin was used as a loading control. Uncropped immunoblots of (**B**) are available in Supplementary File S1.

Subgenomic replicon systems are a standard approach to quantify HCV RNA replication independently of full virion production, and luciferase reporters provide a sensitive, quantitative surrogate for replicon activity in hepatoma cells. Replicon-based assays have been widely used in HCV biology and antiviral development because they isolate replication-dependent processes and enable precise measurement of changes in replication following genetic or pharmacologic perturbations [49,50]. Given that HCV can enhance glycolysis and that virus-induced metabolic remodeling can support persistence and amplification, it is biologically plausible that HKDC1-driven glycolysis contributes directly to replicon fitness. To explore the effect of HKDC1 on HCV replication, Renilla luciferase activity was measured in HCV-positive APC140 cells expressing either non-targeting or HKDC1-targeting shRNA. Depletion of HKDC1 resulted in a significant reduction in Renilla signal relative to controls (Figure 6A). This reduction was observed consistently across replicate experiments, confirming that loss of HKDC1 markedly lowers replicon-dependent Renilla expression. In addition, the impact of HKDC1 depletion on HCV protein expression was also assessed. Immunoblot analysis showed reduced NS5B protein expression in APC 140 cells expressing HKDC1 shRNA compared with cells transduced with a non-targeting shRNA control (Figure 6B). This suggests that HKDC1 knockdown reduces HCV replication.

## 4. Discussion

Chronic HCV infection rewires host metabolism to sustain viral persistence and disease progression. Prior work has established that HCV non-structural proteins, particularly NS5A, enhance glycolysis through direct modulation of hexokinase activity, including binding-dependent activation of HK2 and glucokinase [11,12]. While these pathways highlight important mechanisms by which HCV rewires glucose metabolism, they do not fully explain how total hexokinase activity is increased in HCV-associated hepatocellular carcinoma, particularly in the absence of consistent HK2 induction. Our data extend this framework by identifying HKDC1 as a distinct, isoform-selective driver of glycolytic enhancement in the HCV replicon context.

In this study, we identified HKDC1 as a virus-activated metabolic mediator that links HCV replication with sustained glycolytic remodeling in hepatoma cells. Identifying this role of HKDC1 clarifies how a single host enzyme can support both the energy and biosynthetic demands of viral replication and the metabolic features associated with liver cancer progression. We showed that HCV-positive APC140 cells exhibited increased total hexokinase activity and elevated pyruvate and lactate levels, consistent with enhanced glycolytic flux. In addition, HKDC1 was selectively induced and enriched in the cytoplasm. HKDC1 knockdown reduced glycolytic output without compensatory changes in other hexokinase isoforms while dampening replicon activity. These observations support a model in which HKDC1 maintains a glycolytic state required for ongoing replication, rather than solely at initiation. Prior work has highlighted the NS5A-mediated enhancement of glycolysis via direct effects on HK2 [11,12]. Moreover, chronic HCV infection promotes mitochondrial dysfunction, oxidative stress, and stabilization of hypoxia-inducible factor-1α, which together augment glycolytic gene expression and metabolic flux [51].

HKDC1 has emerged as an important regulator distinct from classical hexokinases in recent cancer metabolism research. Unlike HK1 and HK2, which are widespread in the tumor microenvironment, HKDC1 is enriched in the malignant cell compartment and has been reported to promote migration and invasion in hepatocellular carcinoma through distinct metabolic effects. In HCC and cholangiocarcinoma models, HKDC1 overexpression alters tricarboxylic acid cycle metabolites, is associated with metastasis potential, and is induced under hypoxic conditions, where it can stabilize β-catenin to promote stemness [32]. Although the present study did not directly assess migratory or invasive behavior, the identification of HKDC1 as an HCV-responsive metabolic mediator suggests that viral induction of HKDC1 may contribute to metabolic programs associated with tumor aggressiveness. Functional migration and invasion studies are warranted in the future to directly define this relationship. Additional work has shown that HKDC1 interacts with mitochondrial proteins at the outer mitochondrial membrane and drives metabolic reprogramming, shifting glucose metabolism away from oxidative phosphorylation and toward aerobic glycolysis [43]. These roles are consistent with our findings that HKDC1 contributes to enhanced glycolytic activity in HCV-infected HCC cells. Consistent with these observations, our findings demonstrate that HKDC1 is selectively induced in HCV-positive hepatoma cells and that its depletion significantly reduces total hexokinase activity and downstream glycolytic metabolite production. The absence of compensatory changes in HK1–HK4 following HKDC1 knockdown strengthens the conclusion that HKDC1 is the predominant contributor to elevated glycolytic flux in this setting.

Subcellular localization provides additional support for the functional role of HKDC1 in this context. HKDC1 was localized predominantly to the cytoplasm in both Huh7.5.1 and APC140 cell lines, which is consistent with its established cytoplasmic–mitochondrial distribution. In addition, the cytoplasmic HKDC1 levels were higher in HCV-positive APC140 cells, suggesting that viral infection enhances HKDC1 abundance or stability in this compartment. This cytoplasmic enrichment may enhance glucose phosphorylation, promote glycolytic flux, and support metabolic flexibility, thereby facilitating proliferation and survival within the virus-altered tumor microenvironment. This is consistent with the observed increases in total hexokinase activity and glycolytic end products. Such compartmental redistribution has been recognized as a critical mechanism by which viruses and oncogenic signals optimize metabolic enzyme function, and our data suggest that HCV exploits this strategy through HKDC1. HKDC1 also showed a punctate cytoplasmic staining pattern, which aligns with previous findings suggesting its association with mitochondria. This pattern indicates a potential spatial organization that may facilitate the coupling of glucose phosphorylation to mitochondrial metabolism, especially during viral infections and metabolic stress.

Loss-of-function experiments underscore a causal link between HKDC1 and both metabolic and virological outcomes. HKDC1 knockdown reduces total hexokinase activity as well as extracellular pyruvate and lactate, and significantly lowers the Renilla signal in the subgenomic replicon system. The absence of compensatory induction of other hexokinase isoforms argues that the observed metabolic and virologic effects stem directly from HKDC1 depletion. Because replicon-dependent luciferase expression reflects viral RNA replication, these results indicate that HKDC1-driven glycolytic remodeling directly supports HCV replication. This finding aligns with broader evidence that viral replication is tightly coupled to the host metabolic state and underscores the functional importance of glucose metabolism for HCV fitness. In addition to reducing replicon-dependent luciferase activity, HKDC1 depletion also reduces the abundance of the HCV non-structural polymerase NS5B. Because NS5B is essential for viral RNA replication and is produced in proportion to active replicon function, decreased NS5B protein levels provide independent biochemical support for impaired HCV replication following HKDC1 knockdown. This finding strengthens the interpretation that the reduction in the Renilla signal reflects a true defect in viral replication rather than a nonspecific effect on reporter expression or cell viability.

The mechanism by which HCV upregulates HKDC1 remains to be fully elucidated. Potential drivers include viral non-structural proteins (such as NS5A and NS5B) and host stress pathways, such as HIF-1α, FoxO1, or AMPK/mTOR signaling [51]. Prior work has highlighted NS5A-mediated enhancement of glycolysis via direct effects on HK2 [11,12]. NS5A has been shown to activate glycolytic enzymes and lipogenesis, partly through direct interactions and modulation of transcriptional programs, suggesting that virus–host protein interactions may trigger HKDC1 induction [12]. Although the expression of viral proteins, especially NS5A and NS5B, correlates with elevated HKDC1 levels in the replicon model, whether individual viral proteins directly regulate HKDC1 expression remains poorly understood and will be the focus of future mechanistic studies. It is also possible that chronic infection-associated hypoxia and oxidative stress contribute to HKDC1 upregulation via hypoxia-responsive elements, as seen in cancer models [32]. Systematic testing using different viral genotypes and pathway-specific perturbations will clarify whether HKDC1 is transcriptionally induced, post-translationally stabilized, or relocalized by these cues.

The implications of our study for liver cancer biology are notable. Enhanced glycolysis and lactate production are hallmarks of cancer metabolism that support anabolic growth, immune modulation, and tumor progression. Elevated lactate can acidify the microenvironment, inhibit antitumor immune responses, and promote angiogenesis, all of which contribute to tumorigenesis. The specific induction of HKDC1 by HCV suggests that this enzyme may serve as a mechanistic bridge connecting chronic viral infection to oncogenic metabolic reprogramming. Moreover, high HKDC1 expression has been associated with poor prognosis and aggressive tumor phenotypes in HCC patients, reinforcing its relevance in liver cancer biology [34]. By positioning HKDC1 as a bridge between chronic HCV replication and glycolytic reprogramming, our data suggest that HKDC1 serves as both a mechanistic node and a potential therapeutic target in HCV-associated HCC.

Although our study provides clear evidence that HKDC1 is selectively induced by HCV subgenomic replicons and drives metabolic reprogramming, there are inherent limitations. The use of subgenomic replicons mimics viral RNA replication but does not capture viral assembly or the full spectrum of HCV infection. Experiments were performed using hepatoma cell lines, which may not fully reflect the metabolic complexity of primary hepatocytes in vivo. Future studies in primary hepatocytes and animal models, validation in additional HCV genotypes, and dissection of viral protein–HKDC1 signaling will be essential to validate the broader relevance of these findings and to define the upstream regulatory pathways that control HKDC1 induction during infection.

## 5. Conclusions

In conclusion, our study identified HKDC1 as a novel mediator of HCV-induced metabolic reprogramming, which may have significant implications for HCC development. HKDC1 enhances glycolytic activity, supporting HCV replication and creating metabolic conditions conducive to HCC progression. These findings underscore the importance of virus–host metabolic interactions in the pathogenesis of HCC and suggest that targeting HKDC1 or its downstream metabolic pathways could provide new strategies for the prevention or treatment of HCV-associated liver cancer.

## Data Availability

The data supporting this study’s findings are available from the corresponding author upon reasonable request.

## Supplementary Materials

The following supporting information can be downloaded at https://www.mdpi.com/article/10.3390/v18040423/s1. File S1: Original blots (uncropped).

## Abbreviations

The following abbreviations are used in this manuscript:

HCV	Hepatitis C virus
DAPI	4′,6-diamidino-2-phenylindole
GAPDH	Glyceraldehyde-3-phosphate dehydrogenase
GCK	Glucokinase
HCC	Hepatocellular carcinoma
HK	Hexokinase
HKDC1	Hexokinase domain-containing protein 1
NADP	Nicotinamide adenine dinucleotide phosphate
NS5A	Non-structural 5A
NS5B	Non-structural 5B
RLUs	Relative light units
SEM	Standard error of the mean
shRNA	Short hairpin RNA
VDAC1	Voltage-dependent anion channel 1
