# Supplementary material for: Hepatitis C Virus (HCV)—Mediated Activation of Hexokinase Domain-Containing Protein 1 (HKDC1) Promotes Hexokinase Activity and Metabolic Reprogramming
Source: Viruses. 2026 Mar 31;18(4):423. doi: 10.3390/v18040423 (PMC13119884; doi:10.3390/v18040423)

Figure S1: Uncropped immunoblots of Figure 1B.

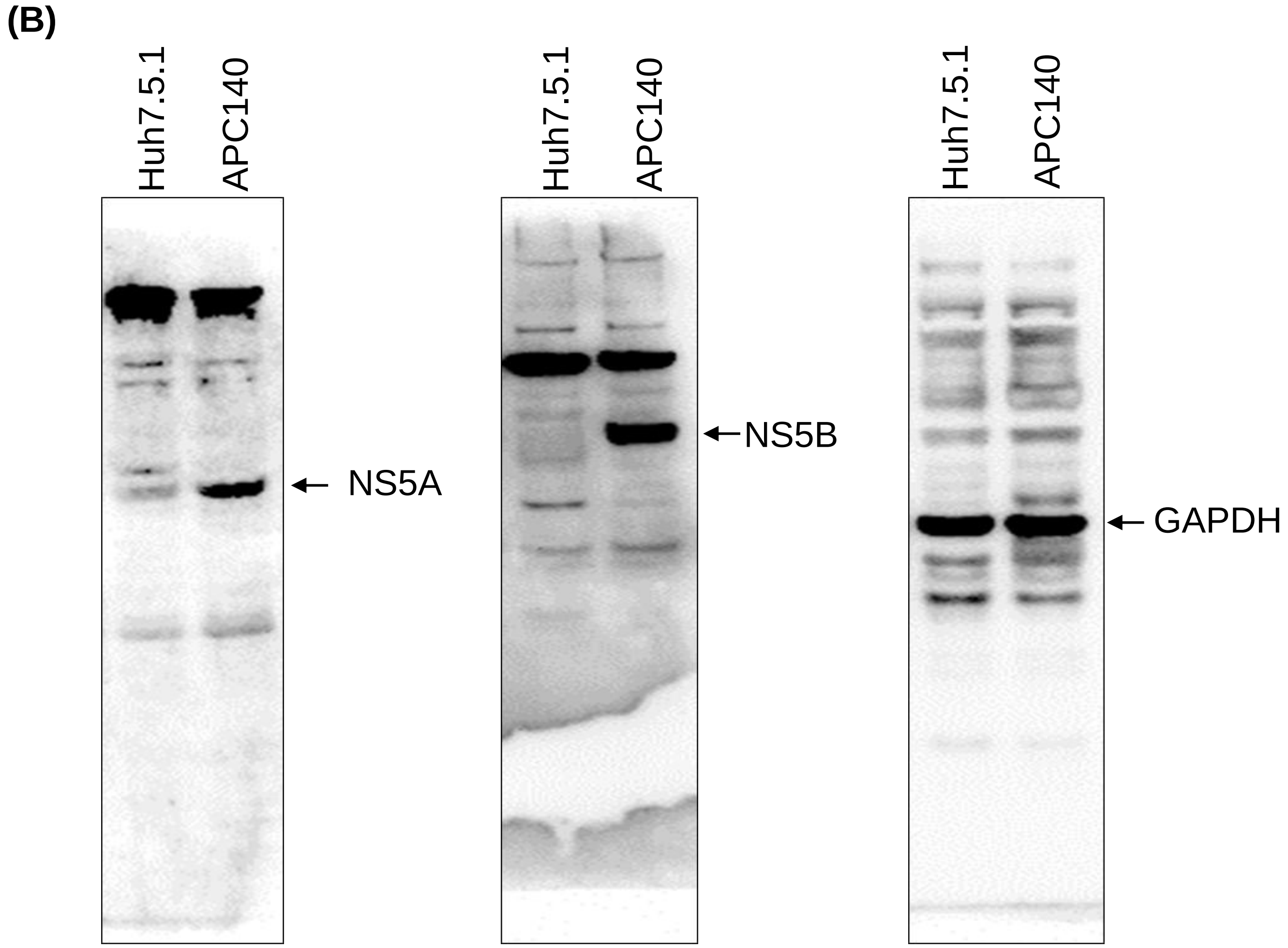

Figure S2: Uncropped immunoblots of Figure 2B.

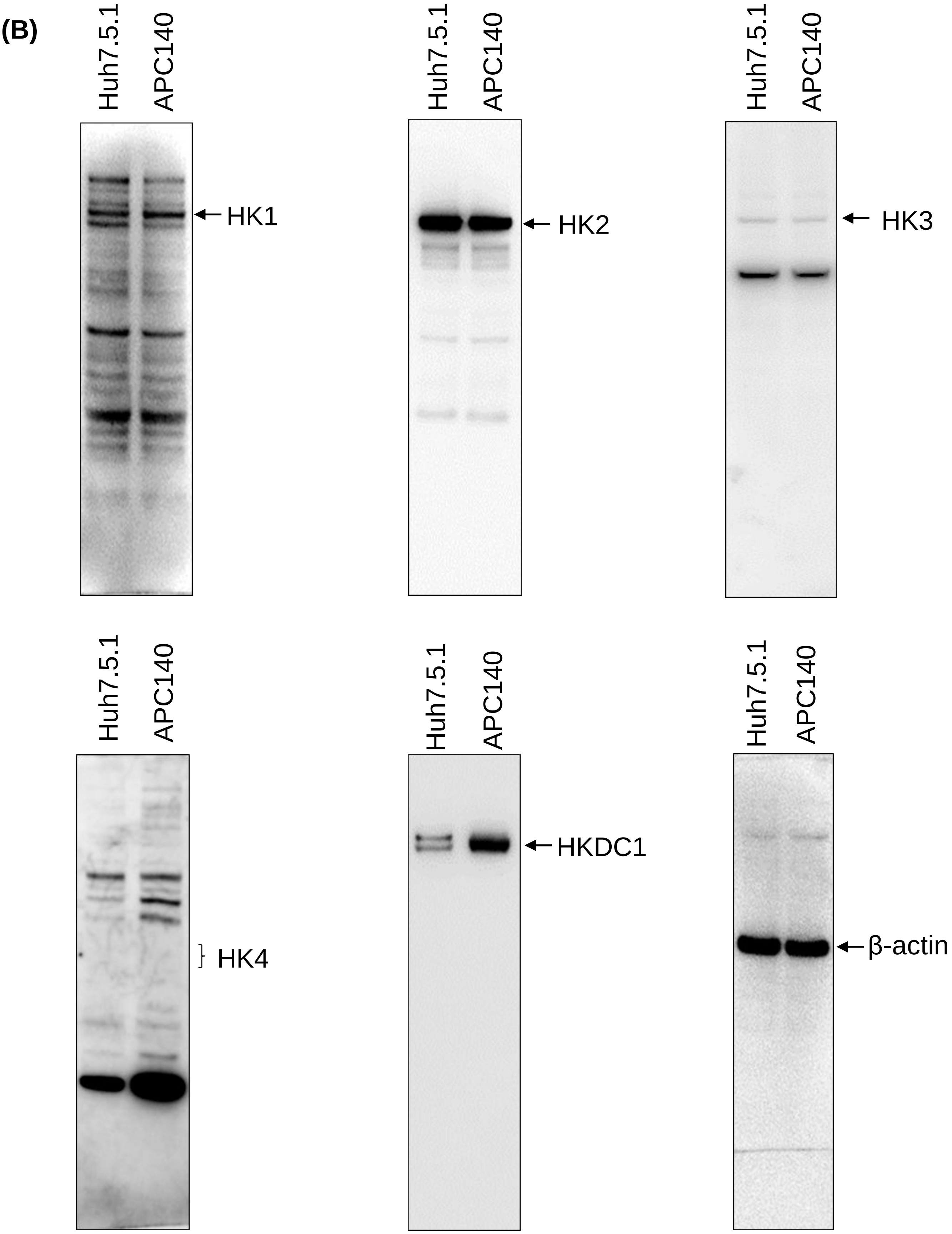

Figure S3: Uncropped immunoblots of Figure 3A.

(A)

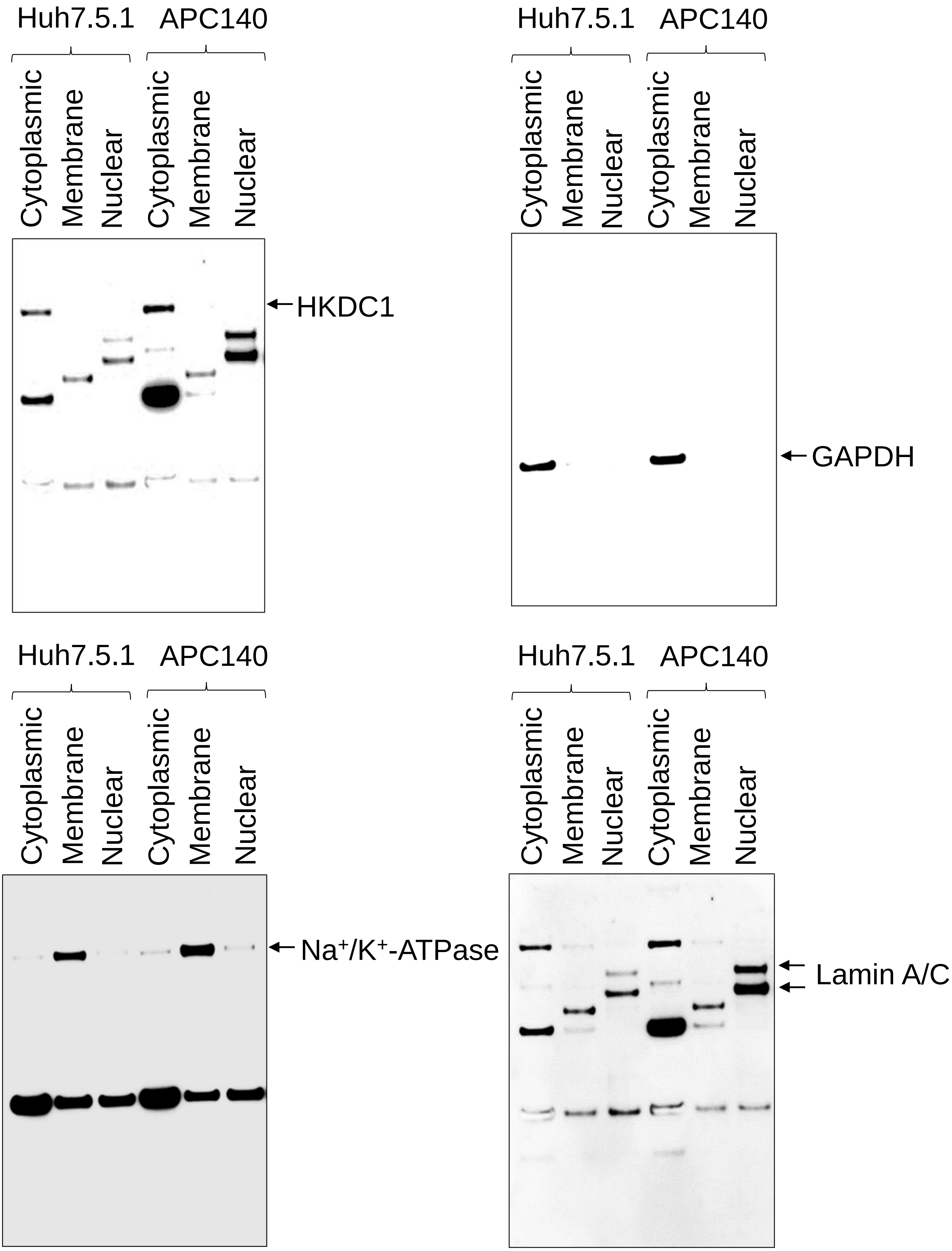

Figure S4: Uncropped immunoblots of Figure 4B.

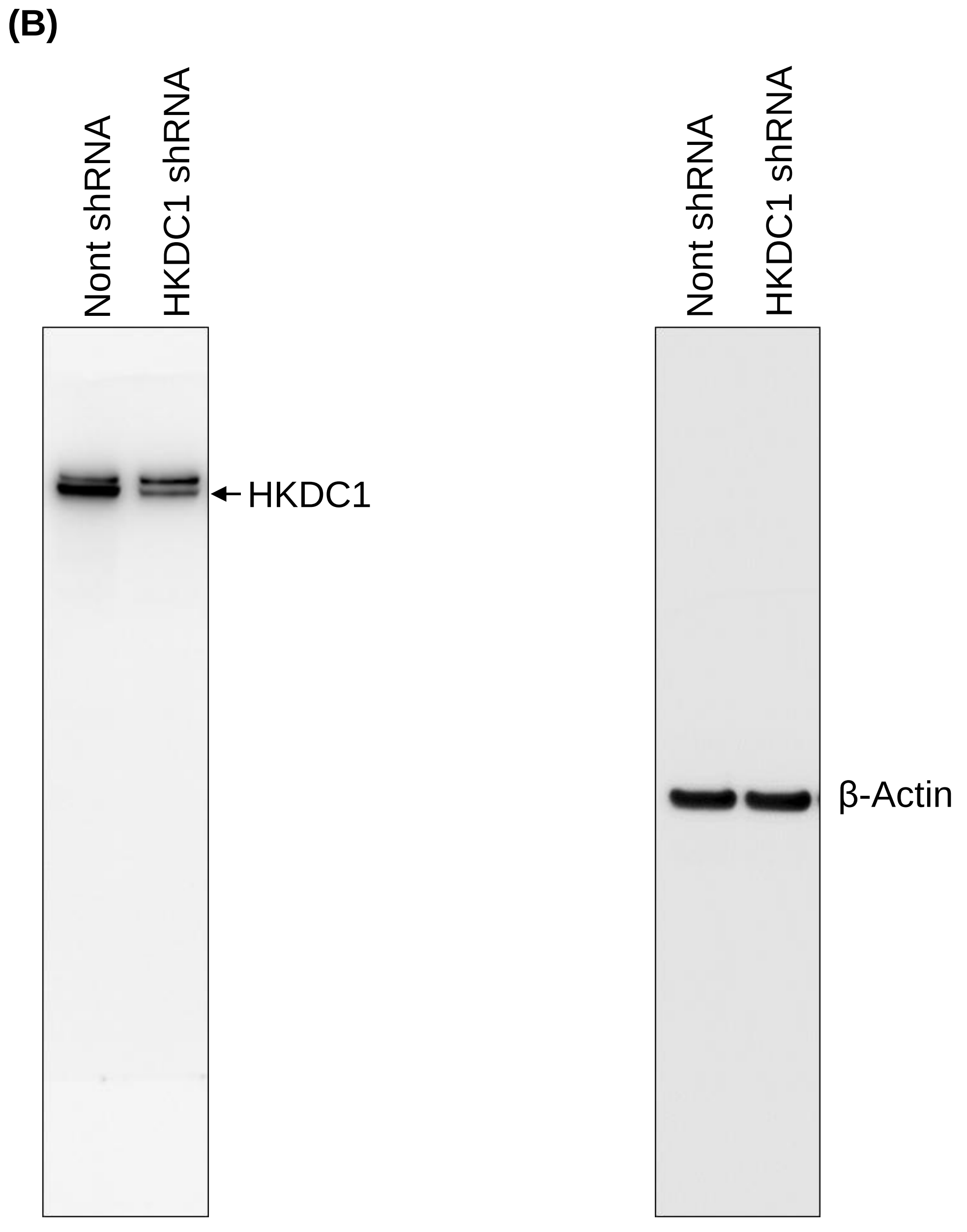

Figure S5: Uncropped immunoblots of Figure 5B.

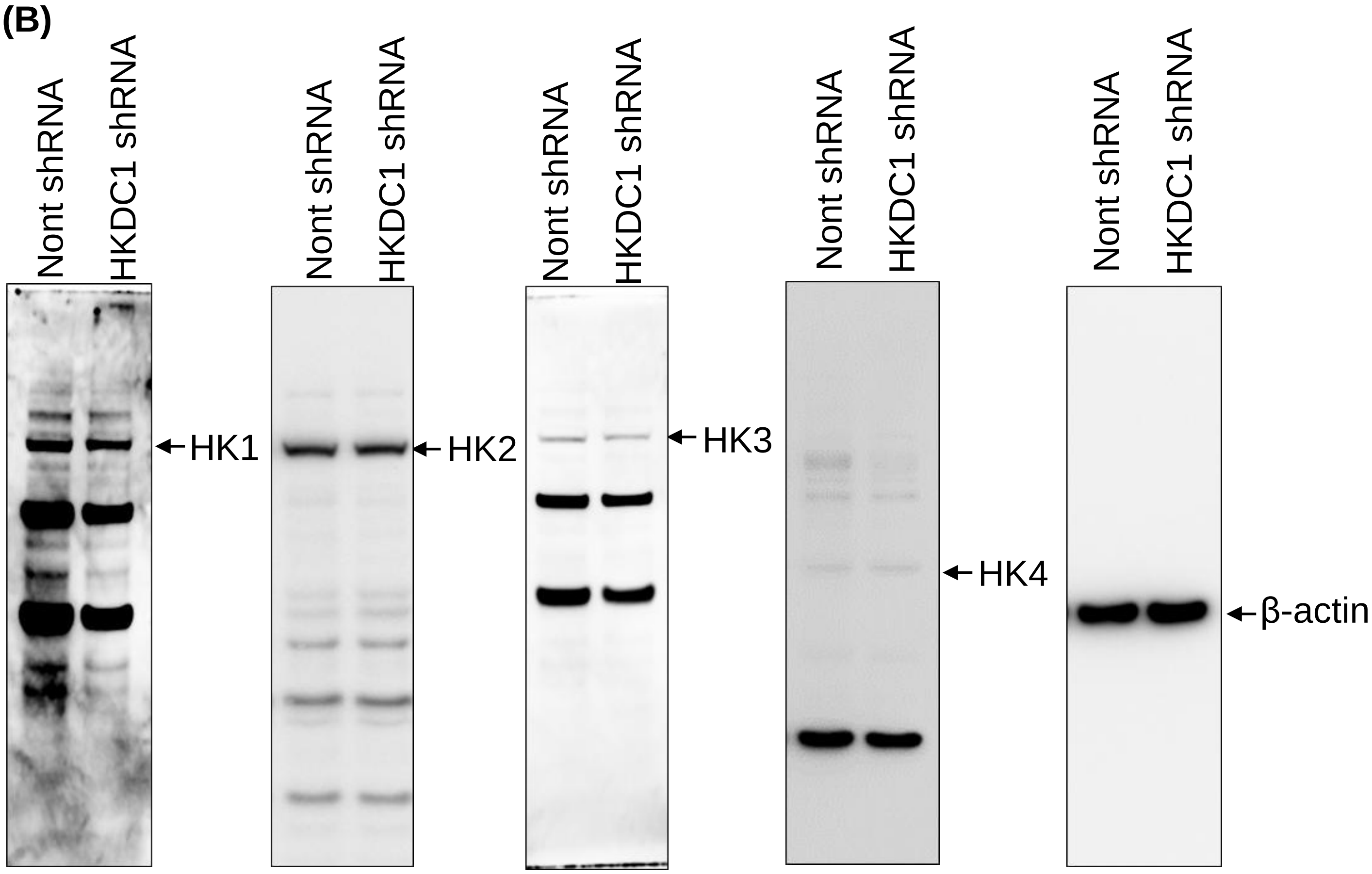

Figure S6: Uncropped immunoblots of Figure 6B.

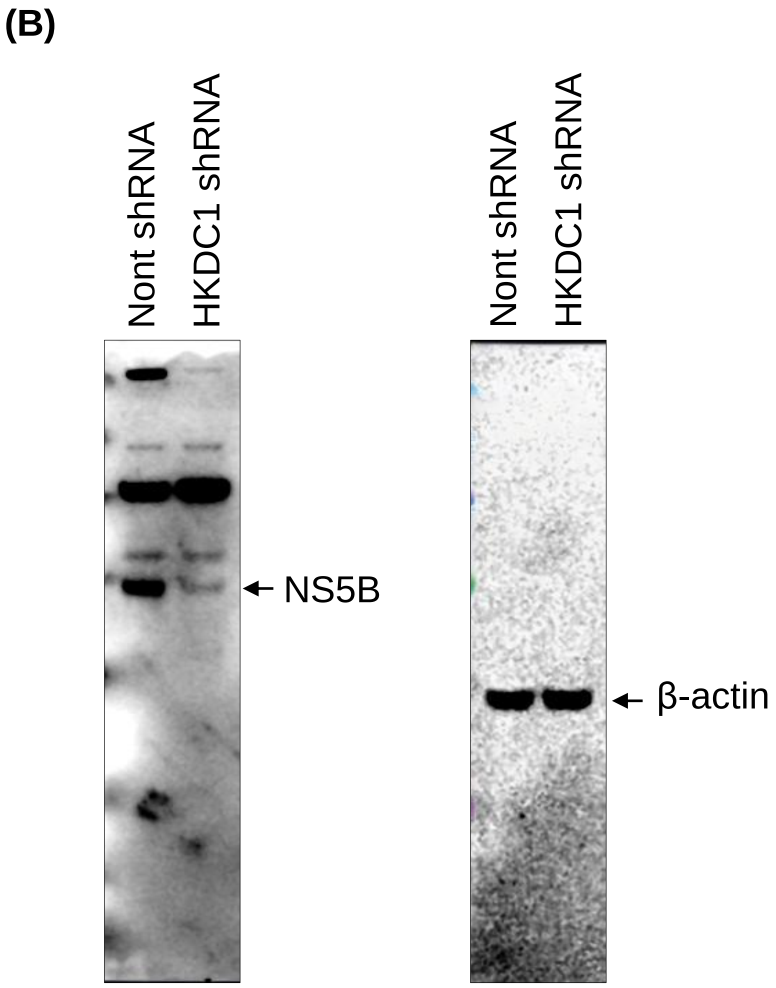

Supplement: Supplementary file 1 [file viruses-18-00423-s001.zip › viruses-4198657-supplementary.pdf]
